# MAPPING SEDENTARY BEHAVIOUR USING WEARABLE DEVICES AND DIARIES IN OLDER ADULTS WITH FRAILTY: A FEASIBILITY STUDY

**DOI:** 10.1093/geroni/igad104.3463

**Published:** 2023-12-21

**Authors:** Isabel Rodrigues, Jonathan Adachi, Steven Bray, Qiyin Fang, Dylan Kobsar, Alexander Rabinovich, Rong Zheng, Alexandra Papaioannou

**Affiliations:** McMaster University, Hamilton, Ontario, Canada; McMaster University, Hamilton, Ontario, Canada; McMaster University, Hamilton, Ontario, Canada; McMaster University, Hamilton, Ontario, Canada; McMaster University, Hamilton, Ontario, Canada; McMaster University, Hamilton, Ontario, Canada; McMaster University, Hamilton, Ontario, Canada; McMaster University, Hamilton, Ontario, Canada

## Abstract

Older adults with frailty are sedentary. Prior interventions to reduce sedentary time in older adults have not been successful as there is little research about context (posture, location, purpose, social environment). There is limited evidence on feasible measures to assess context of sedentary behaviour in older adults. Our aim was to determine feasibility of measuring context of sedentary behaviour in older adults with frailty using objective and self-report measures. We defined “feasibility process” using recruitment (20 participants within two-months), retention (85%), and refusal (20%) rates and “feasibility resource” if the measures capture context and can be linked (e.g., sitting-kitchen-eating-alone), and are all participants willing to use the measures. Context was assessed using a wearable sensor for posture, indoor positioning system [IPS] for location, and electronic or hard-copy diary for purpose and social-context over three days in winter and spring. We approached 80 individuals, and 58 expressed interest; of the 58, 37 did not enroll due to lack of interest or medical mistrust (64% refusal). We recruited 21 older adults (72±7.3 years, 13 females, 13 frail) within two months and experienced two dropouts (90% retention). The measures captured one domain of context, but the hard copy was not completed with detail making it challenging to link with the other devices. Not all participants were willing to use the wearable devices or electronic diary; but we linked the measures of those who did. Future studies will need to determine the most feasible and valid method to assess the context of sedentary behaviour.

